# Epigenetic Regulation of Neural Transmission after Cerebellar Fastigial Nucleus Lesions in Juvenile Rats

**DOI:** 10.1007/s12311-021-01264-5

**Published:** 2021-04-08

**Authors:** Simeon O. A. Helgers, Svilen Angelov, Marc A. N. Muschler, Alexander Glahn, Shadi Al-Afif, Yazeed Al Krinawe, Elvis J. Hermann, Joachim K. Krauss, Helge Frieling, Kerstin Schwabe, Mathias Rhein

**Affiliations:** 1 Department of Neurosurgery, Hannover Medical School, Hannover, Germany; 2 Department of Psychiatry, Laboratory of Molecular Neuroscience, Social Psychiatry and Psychotherapy, Hannover Medical School, Hannover, Germany

**Keywords:** GAD1, OXTR, Oxytocin, Cerebellum, Methylation, Cerebellar cognitive affective syndrome

## Abstract

Structural and functional abnormalities in the cerebellar midline region, including the fastigial nucleus, have been reported in neuropsychiatric disorders, also comprising the cerebellar cognitive affecting syndrome. In rats, early fastigial lesions reduce social interaction during development and lead to cognitive and emotional deficits in adults, accompanied by compromised neuronal network activity. Since epigenetic mechanisms are implicated in the etiology of neuropsychiatric disorders, we investigated whether fastigial nucleus lesions in juvenile rats would impact epigenetic regulation of neural transmission. The fastigial nucleus was lesioned bilaterally in 23-day-old male rats. Sham-lesion and naïve rats served as controls. DNA methylation was investigated for target genes of the GABAergic, dopaminergic, glutamatergic and oxytocinergic systems in brain regions with anatomic connections to the fastigial nucleus, i.e., medial prefrontal cortex, nucleus accumbens, striatum, thalamus, and sensorimotor cortex. Protein expression was examined for the respective target genes in case of altered DNA methylation between lesion and control groups. Lesioning of the fastigial nucleus led to significant differences in the epigenetic regulation of *glutamate decarboxylase 1* and the *oxytocin receptor* in the nucleus accumbens and the prefrontal cortex. No differences were found for the other target genes and brain regions. Our findings indicate that epigenetic dysregulation after lesioning of the fastigial nucleus may influence long-term recovery and the emergence of behavioral changes. Together with previous behavioral and electrophysiological investigations of this rat model, these observations can play a role in the cerebellar cognitive affective syndrome and other neuropsychiatric disorders.

**Supplementary Information:**

The online version contains supplementary material available at 10.1007/s12311-021-01264-5.

## Introduction

The cerebellar midline region, including the fastigial nucleus, has been recognized to be critically involved in the cerebellar cognitive affective syndrome, a heterogeneous clinical condition described after cerebellar injuries in adults concerning the cognitive-associative, visuospatial, and affective domain [1, 2]. This anatomical region is also discussed in the context of the cerebellar mutism syndrome, which is observed in children after posterior fossa midline tumor resection, and certain neuropsychiatric disorders, including autism spectrum disorders and schizophrenia [3–5].

Anatomically, the cerebellar fastigial nucleus has widespread connections throughout the brain [6]. Projections to the thalamus, the ventral tegmental area, and the nucleus accumbens (NAc) serve as essential relay stations which give rise to disynaptic connections to the prefrontal cortex (PFC) and the striatum [7]. Its function has been associated with motor-related tasks and modulatory effects in the cognitive and affective domains [6–8]. Especially for the non-motor tasks, the anatomical connections of the fastigial nucleus to the NAc and the PFC are of major importance [7–10].

Fastigial nucleus lesions in juvenile rats lead to reduced social interaction during development, accompanied by mild cognitive and affective deficits in adulthood [11, 12]. Furthermore, neuronal activity in the prefrontal cortex was altered in rats with lesions of the fastigial nucleus [12]. However, the effects of early fastigial nucleus lesions on molecular mechanisms in anatomically connected brain regions are not well known.

Epigenetic mechanisms influence cerebral function, and disturbance may lead to aberrant development and ultimately neuropsychiatric dysfunction [13–16]. Epigenetic remodeling is subject to a permanent adaptation influenced by environmental and psychosocial factors [15]. DNA methylation is one of the critical factors for regulation of DNA activity. Such regulative methylation occurs at a cytosine base in immediate proximity to a guanine base (CpG motif). DNA methylation of specific CpG positions with co-localized transcription factors can influence protein expression. In animal models, it has been shown that even minor traumatic brain injuries cause persistent changes in DNA methylation and thereby affect the long-term recovery of these animals [17, 18]. Therefore, lesions of the cerebellar midline structures may cause DNA methylation changes and altered network function.

In the current study, we examined the epigenetic regulation of five target genes, highly implicated in the overall regulation of neural transmission (GABAergic, glutamatergic, dopaminergic system) and more specifically associated with neuropsychiatric disorders, such as autism spectrum disorder (oxytocinergic system), after juvenile lesion of the fastigial nucleus in rats. Analysis was conducted in brain regions with strong anatomic connections to the fastigial nucleus, i.e., the PFC, NAc, the striatum, the thalamus, and the cortex. Targets were chosen across different neurotransmitter systems: *Glutamate decarboxylase 1* (Gad1), the rate-limiting enzyme for the synthesis of GABA, and the primary inhibitory transmitter for intra- and interregional neuromodulation [19]. The *glutamate receptor subunit zeta-1* (GluN1 encoded by the Grin1 gene) of the NMDA receptor, representing the major excitatory transmitter system in the brain, also crucially involved in synaptic plasticity [8, 20]. The *dopamine receptor D2* (Drd2) and *dopamine transporter* (Slc6a3) as targets for the main modulatory transmitter for projections to the NAc and the PFC within the mesocortical and mesolimbic system [21, 22]. And lastly, the *oxytocin receptor* (Oxtr) because of its pivotal role in social function and autism spectrum disorders [23–25]. DNA methylation data was supplemented by protein expression analysis and discussed in relation to behavioral data previously published in the same animal model [11, 12].

## Methods

### Animals

Male Sprague–Dawley (SD) rats (*n* = 38, 56–82 g) were obtained from 13 litters (Charles River Laboratories). Animals were separated from their mothers on postnatal day (PND) 21. Four animals (two pairs of different experimental groups) were housed together in Makrolon Type IV open cages under controlled environmental conditions (22 ± 2 °C; 55 ± 10% humidity; 10/14-h dark/light cycle with lights on at 06:00 a.m.). Until a bodyweight of 200 g, animals were fed ab libitum with standard rodent chow (Altromin, Lage, Germany). Then, 14-g chow was fed per day and animal. Animals always had access to tap water. To ensure the well-being of all animals, bodyweight and clinical scores were assessed regularly, at least two times per week.

All experiments were carried out per the EU directive 2010/63 and were approved by the local animal ethics committee (Lower Saxony State Office for Consumer Protection and Food Safety; AZ 17/2583). All efforts were made to minimize the number of animals used and their suffering.

### Study Design

**Fig. 1:**
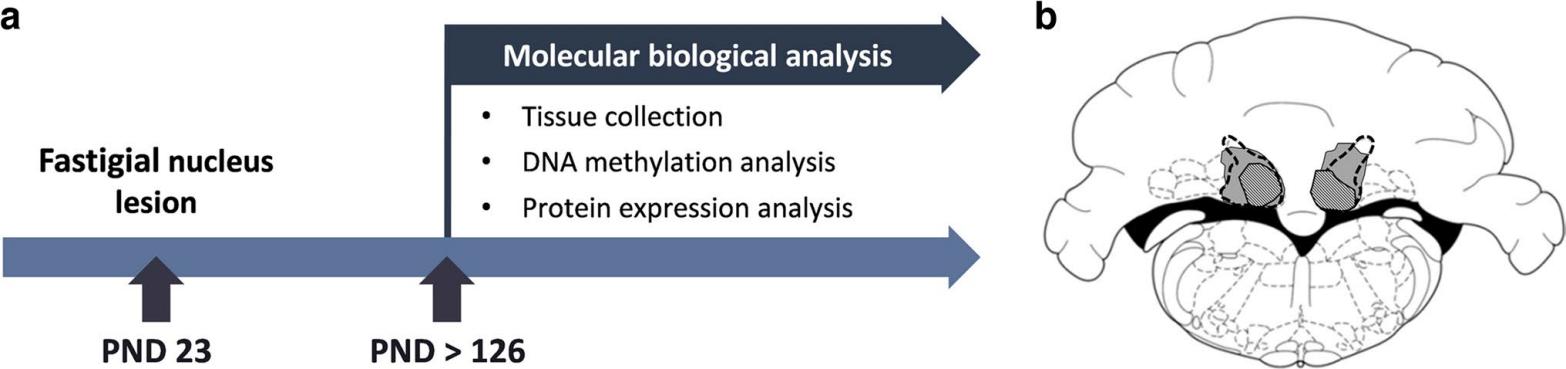
**a** The study design. **b** Sizes of fastigial nucleus lesions after bilateral thermocoagulation. The extent of the smallest lesion (indicated by striped area), the largest lesion (shown in gray), and the boundaries of the fastigial nucleus (indicated by black dashed lines) are depicted on a schematic drawing of a coronal section from the atlas of Paxinos and Watson [26]

Rats were randomly assigned to the lesion (*n* = 17), sham lesion (*n* = 11), or naïve group (*n* = 10). On PND 23 animals of the lesion group were subjected to bilateral stereotaxic lesioning of the fastigial nucleus via thermocoagulation. Rats of the sham lesion group were subjected to the same surgery, but electrodes were only inserted above the fastigial nucleus, and no current was applied**.** As adults (PND 70), parts of the animals (lesion *n* = 9; sham lesion *n* = 10; naïve *n* = 8) were subjected to behavioral testing, as reported in Helgers et al. (2020). As no difference was found between behaviorally tested and not tested rats, animals were grouped for final analysis. At the end of the experiment (PND > 126), brain tissue of different target regions (PFC, NAc, striatum, thalamus, and sensorimotor cortex) was collected and analyzed for epigenetic regulation of different target genes (Gad1, Oxtr, Drd2, Slc6a3, Grin1; see Fig. 1a). Protein expression was investigated if methylation data showed significant differences.

### Surgery

For lesioning of the fastigial nucleus, rats were anesthetized with chloral hydrate (360 mg/kg) with additional local anesthesia at the surgical field (2% xylocaine, AstraZeneca GmbH, Wedel, Germany) and systemic analgesia (Carprofen, 5 mg/kg intraoperatively, 2.5 mg/kg postoperatively for 2 days). The surgical procedure was performed as described in Al-Afif et al. (2019) and Helgers et al. (2020). In brief, the rat’s skull was fixed in a stereotaxic frame with bregma and lambda aligned on the same transversal plane. Above the target nucleus (in relation to lambda: anterior/posterior: − 2.5/ − 3.0; lateral ± 1, ventral − 6.4; Paxinos and Watson [26]), burr holes were drilled, and a monopolar electrode was carefully inserted. Electrical current was applied for 60 s with 300 µA. After that, the electrode was removed, and the surgical wound was closed by suture clips. The surgical procedure for the sham-lesion rats was identical. However, the electrode was only inserted 1 mm above the nucleus, and no current was applied.

### Tissue Collection and Histology

Rats were anesthetized with carbon dioxide and decapitated. Brains were removed and immediately cooled using a freezing microtome stage. Target brain areas were bilaterally collected and snap-frozen in Eppendorf tubes. Brain samples were stored at − 80 °C until further processing. The cerebellum was fixed with 4% paraformaldehyde for 24 h. After immersion in 30% sucrose for another 24 h, brain sections of 50 µm thickness were obtained using a cryostat. The correct location of the fastigial nucleus lesion was bilaterally verified and quantitatively determined in Nissl-stained sections (thionine-staining) using a Zeiss light microscope. The absence of lesions was verified in sham-lesion rats (see Fig. 1b).

### Tissue Processing

According to the manufacturer’s recommendations, DNA, RNA, and protein extraction were performed using the AllPrep Kit (QIAGEN AG, Hilden, Germany). We adapted the last step of the protein purification as follows: After washing the precipitated protein with 70% Ethanol (step 16) and drying of the pellet (step 17), the protein pellet was dissolved using a chaotropic buffer (Promelt; MoBiTec, Göttingen, Germany). The samples were then incubated at 95 °C for 5 min to ensure complete dissolution of the pellet, followed by storage at − 80 °C.

### DNA Methylation Analysis

**Fig. 2:**
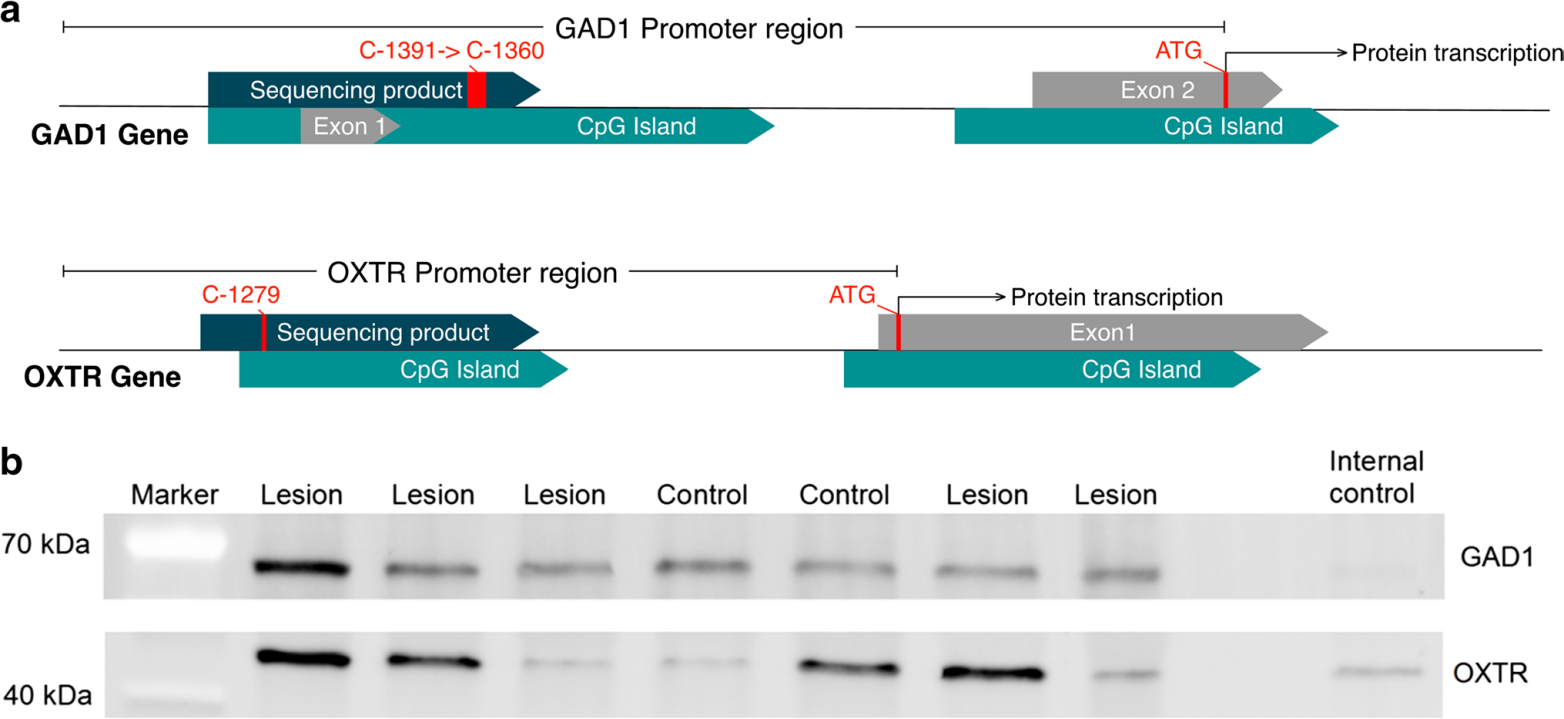
**a** Map of the Gad1 and the Oxtr promoter. CpG islands, exons, and protein transcription start (ATG) are indicated in the schematic drawing. Furthermore, the sequencing product and the specific CpG positions with altered methylation are indicated. **b** Example western blots for Gad1 and Oxtr. Because of sample randomization, not all experimental groups are displayed. The internal control sample was applied to every gel for normalization

According to the manufacturer’s recommendations, DNA samples were bisulfite-converted using the EpiTects 96 Bisulfite Kit (QIAGEN AG, Hilden, Germany). The concentration and purity of the DNA samples were determined via a Denovix DS11 spectrophotometer (DeNovix Inc., Wilmington, USA). Target regions were subjected to a touch-down PCR using HotStarTaq Master Mix Kit (Qiagen, Hilden, Germany). Primers were designed to cover CpG-islands preceding the protein transcription start site in the highly regulatory promoter region of each gene using the Software Geneious R11 (Biomatters Ltd., Auckland, New Zealand; see Fig. 2a for GAD1 and OXTR). For primer sequences and fragments, see Suppl. Table 1. PCRs were performed on a C1000 Thermal Cycler (BIO-RAD, Hercules, CA, USA). For purification of the PCR product, Agencourt AMPure XP beads (Beckman Coulter, Brea, USA) were used on a Biomek NxP pipetting platform (Beckman Coulter, Brea, USA).

Sequencing of the target genes was performed with the BigDye Terminator v3.1 Cycle Sequencing Kit (ABI Life Technologies, Grand Island, USA) and was used according to the manufacturer’s instructions. Pre-sequencing cleanup was processed using Agencourt CleanSEQ beads (Beckman Coulter GmbH, Krefeld, Germany). The final products were sequenced on an Applied Biosystems 3500xl DNA Analyzer (ABI Life Technologies, Grand Island, USA).

After technical quality assessment of sequences using Sequence Scanner Software (ABI Life Technologies, Grand Island, USA), files were processed using the Epigenetic Sequencing Methylation Analysis Software to determine the methylation rates of the CpG sites covered by the PCR fragment.

### Western Blot Analysis

Protein concentrations were estimated using a Bradford Assay Reagent (Bio-Rad Laboratories, Hercules, USA). For western blotting, 25 µg protein was loaded on 4–20% gradient TGX precast protein gels (Bio-Rad). PageRuler (Thermo Fisher Scientific, Waltham, USA) and an internal control, identical across blots, were applied on each gel. Blotting was done using the Trans-Blot Turbo Kit (Bio-Rad Laboratories, Hercules, USA) on a PVDF membrane according to the manufacturer's recommendations. The membrane was blocked using the EveryBlot buffer (Bio-Rad Laboratories, Hercules, USA). Primary antibodies against GAD1 (GAD-67 Antibody (F-6)—monoclonal sc-28376 labeled with AF488; Santa Cruz Biotechnologies, Dallas, USA), OXTR (anti-oxytocin receptor antibody, ab217212; Abcam, Cambridge, UK) were applied overnight at 4 °C in a dilution of 1:500 in Everyblot buffer. The secondary antibody (goat anti-rabbit IgG, labeled with AF546, A-11035; Thermo Fisher Scientific, Waltham, USA) for the unlabeled OXTR antibody was applied 1 h at room temperature in a dilution of 1:10,000 in EveryBlot blocking buffer. The membrane was washed three times with TBST after incubation with each antibody. Fluorescence signals were detected on a Chemidoc MP multiplex fluorescence detection system (Bio-Rad Laboratories, Hercules, USA).

### Statistical Analysis

Analysis of the data was performed using SPSS 26 (IBM, Armonk, NY, USA) and formatted for display with GraphPad Prism 8 (GraphPad, LaJolla, CA, USA).

Methylation results were assessed for overall quality, only including CpGs with 95% data available and animals with at least 95% complete data points. For western blot normalization, we used a method published elsewhere [27]. Briefly, all values were first normalized to the blot-internal control lane, then divided by the average of the lesion values, resulting in control and sham-lesion mean values being displayed in percental relation to lesion group mean values. To avoid extreme outliers, we limited the protein results to values inside the 1.5 inter-quartile range boundaries. The exclusion of outliers did not alter the main results.

Methylation and protein data were both normally distributed according to the Kolmogorov Smirnoff test, except for methylation data for oxytocin, which was visually inspected and confirmed to be normally distributed. We initially used an ANOVA with Tukey’s correction for multiple testing to compare mean and single CpG methylation and protein level comparisons between treatment groups in each brain region.

Transcription factor prediction was performed using the EMBOSS protein database plugin (http://emboss.open-bio.org/) in Geneious R11 (Biomatters, Auckland, New Zealand) with a minimum matching base count of 7 for GAD1 and 6 for OXTR and allowing for a maximum one base mismatch.

## Results

All rats recovered from surgery within the first postoperative days. Some lesion animals initially showed postural instability, gait ataxia, and coordination deficits. However, one week after surgery, only minor deficits were detectable. Sham-lesion animals showed no motor deficits.

### Histological Analysis

Brains of all animals were examined for the correct location of fastigial nucleus lesions. Only rats with lesions of the fastigial nucleus of more than 50% in both hemispheres accompanied by intact neighboring lesions were included in the final analysis as lesion group (see Fig. 1b). In sham lesion rats, the absence of a fastigial nucleus lesion was verified. For the final analysis, 10 of 17 lesions (59%), all 11 sham lesion, and all 10 naïve control animals were included.

### Methylation of Drd2, Slc6a3, and Grin1 across Experimental Groups

Drd2, Slc6a3, and Grin1 showed no significant differences in methylation levels (see Suppl. Figure 1 and Suppl. Table 2). Therefore, these targets were not used for western blot analysis.

### Gad1 Methylation Differs between Lesion Rats and Control Groups

**Fig. 3:**
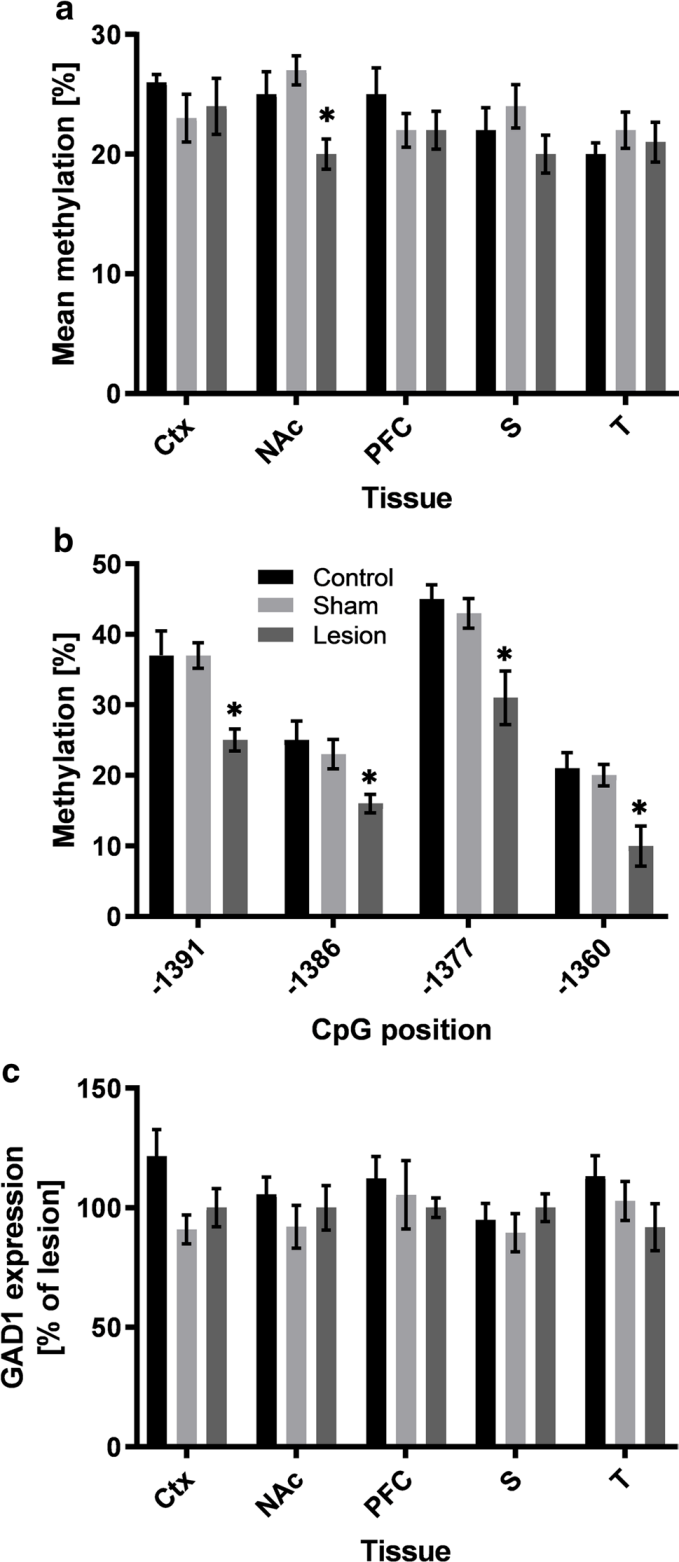
**a** Gad1 mean DNA methylation (%) for all analyzed brain regions. **b** Methylation of specific CpG positions (− 1391, − 1386, − 1377, − 1360) in the NAc. **c** Protein expression (% of lesion animals) for all analyzed brain regions. Data is shown as mean ± SEM for lesion, sham-lesion, and control animals. Significant differences compared to sham-lesion and control animals are shown by an asterisk (*, *p* < 0.05). Abbreviations: Ctx, sensorimotor cortex; NAc, nucleus accumbens; PFC, prefrontal cortex; S, striatum; T, thalamus

In the NAc, the mean methylation of the Gad1 promoter region was significantly different between experimental groups (*F*_2,28_ = 5.394, *p* = 0.010). Post hoc testing showed significantly lower methylation of lesion rats than sham-lesion animals (*p* = 0.009) and a tendency towards lower values between lesion rats and controls (*p* = 0.09, see Fig. 3a). In line with that, four CpGs were identified which displayed hypomethylation in lesion rats compared to sham-lesion and control rats, as shown by a one-way ANOVA (CpG − 1391: *F*_2,28_ = 8.703, *p* = 0.001; CpG − 1386: *F*_2,28_ = 5.163, *p* = 0.013; CpG − 1377: *F*_2,28_ = 7.414, *p* = 0.003; CpG − 1360: *F*_2,28_ = 8.272, *p* = 0.002) and subsequent post hoc testing (all *p* < 0.041; see Fig. 3b). The in silico analysis for existing transcription factor motifs overlapping with respective CpG positions in the promoter region revealed several activating transcription factors potentially affected by methylation. Factors with direct overlap are the CACCC-binding factor (CpG − 1360), E2F-1 (CpG − 1377), SP1 (CpG − 1391, − 1386, − 1377), GR (CpG − 1391, − 1386, − 1377, − 1360), GATA-1 (CpG − 1377), NF-1 (CpG − 1391), TDEF (CpG − 1386), and TBP (CpG − 1360) (see Suppl. Table 3).

For the other brain regions which were examined, no difference in mean methylation was found between the experimental groups (PFC: *F*_2,25_ = 1.040, *p* = 0.368; Ctx: *F*_*2*,24_ = 0.475, *p* = 0.628; S: *F*_2,28_ = 1.531, *p* = 0.234; T: *F*_*2*,27_ = 0.778, *p* = 0.469).

### Oxtr Methylation Differs between Lesion Rats and Control Groups

**Fig. 4:**
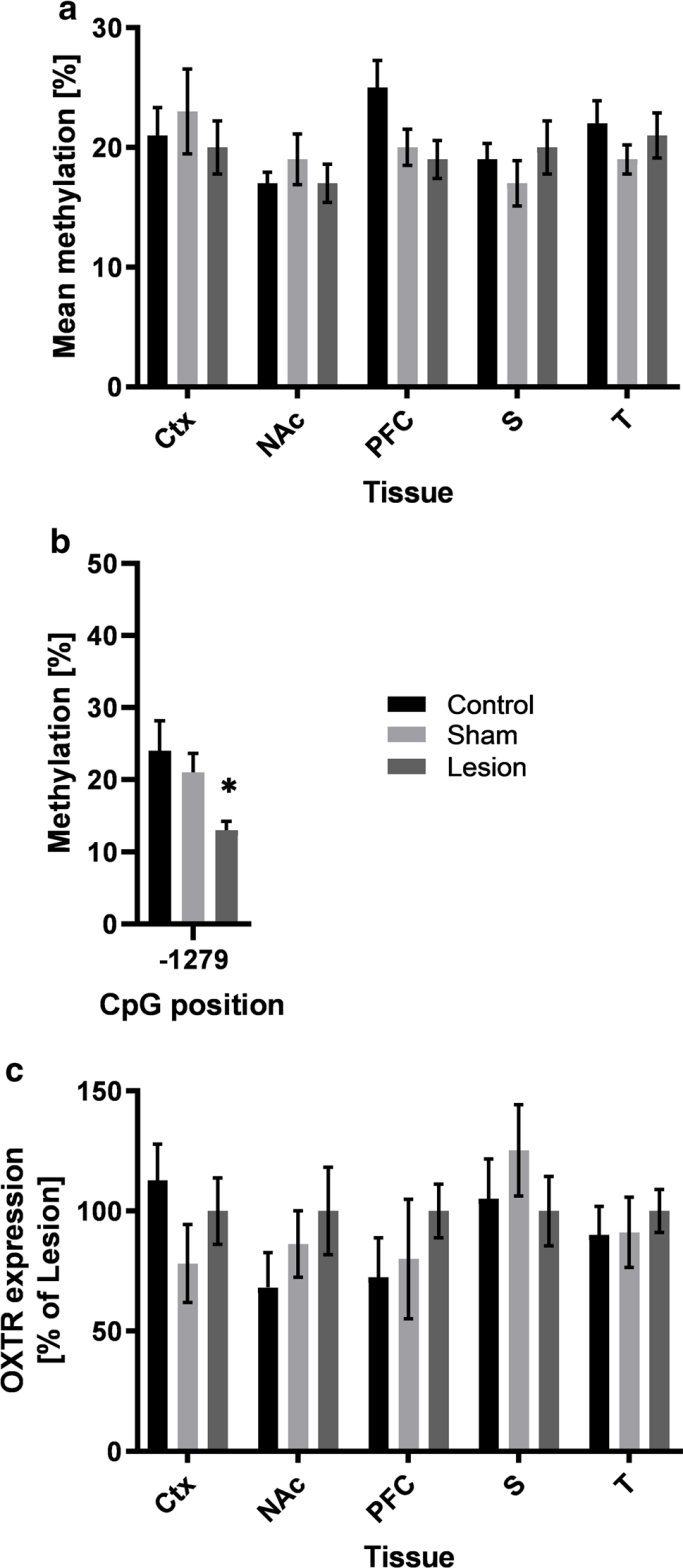
**a** Oxtr mean DNA methylation (%) for all analyzed brain regions. **b** Methylation of specific CpG position (− 1279) in the PFC. **c** Protein expression (% of lesion animals) for all analyzed brain regions. Data is shown as mean ± SEM for lesion, sham-lesion, and control animals. Significant differences between sham-lesion and control animals are indicated by an asterisk (*, *p* < 0.05). Abbreviations: Ctx, sensorimotor cortex; NAc, nucleus accumbens; PFC, prefrontal cortex; S, striatum; T, thalamus

Mean methylation of the Oxtr promotor in all brain regions analyzed was not significantly different between experimental groups (PFC: *F*_2,21_ = 2.516, *p* = 0.105; NAc: *F*_2,28_ = 0.542, *p* = 0.588; Ctx: *F*_2,24_ = 0.298, *p* = 0.745; S: *F*_2,26_ = 0.610, *p* = 0.551; T: *F*_2,28_ = 0.607, *p* = 0.552; see Fig. 4a). However, in the PFC, one CpG position was less methylated in lesion animals compared to control and sham-lesion animals, as indicated by one-way ANOVA (CpG − 1279: *F*_2,28_ = 4.660, *p* = 0.018). Post hoc testing confirmed significantly less methylation in lesion animals as compared to control animals (*p* < 0.021) and a strong tendency between lesion and sham-lesion animals (*p* = 0.059; see Fig. 4b). In silico analysis of the differentially methylated CpG position for existing transcription factor motives revealed a number of activating factors potentially influenced by methylation. Overlapping factors with the CpG − 1279 are SP1, NF-Zc, and E2F-1 (see Suppl. Table 3).

### Expression of Gad1 and Oxtr in Lesion and Control Groups

Western blot analysis of Oxtr and Gad1 showed highly variable expression levels: in some rats increased, but reduced or not altered in others compared to controls. In Gad1, 7 values (lesion: 1; sham lesion: 5; control: 1) and in Oxtr 7 values (control: 1; sham lesion: 6) across tissues were excluded from analysis because of being identified as extreme outliers (i.e., more than 1.5 IQR)**.** Overall, no quantitative differences in protein expression were found between lesion, sham lesion, and control animals (Gat1: all *p* > 0.390; Oxtr: all *p* > 0.371 see Figs. 3c and 4c respectively; see Suppl. Table 2 for detailed results). Example blots for Gad1 and Oxtr are shown in Fig. 2b.

## Discussion

The cerebellar fastigial nucleus has widespread anatomical connections to brain regions involved in motor and non-motor functions [6–8]. In rats, early fastigial lesions reduce social interaction during development and lead to cognitive and emotional deficits in adulthood, accompanied by compromised neuronal network activity. We hypothesized that the behavioral and neuronal disturbances already found after lesions of the fastigial nucleus in juvenile rats [11, 12] would impact epigenetic regulation of neural transmission. The main result was that epigenetic regulation of Gad1 and Oxtr was affected in rats with fastigial nucleus lesions.

Gad1 is the rate-limiting enzyme that converts glutamate to GABA. It accounts for 80–90% of the overall GABA levels in the brain and, therefore, directly influences intra- and interregional inhibitory GABAergic neurotransmission [19]. Epigenetic regulation of the Gad1 promoter has already been shown to alter Gad1 expression levels with direct consequences for the balance between excitation and inhibition in brain regions involved in certain neuropsychiatric disorders such as autism, schizophrenia, and panic disorders [28–31].

In the current study, we found hypomethylation of the Gad1 promoter in the NAc of rats with lesions of the cerebellar fastigial nucleus. Together with the in silico results for transcription factor predication, the lower methylation of activating factors portends higher protein expression. Even though this relation could not be observed in correlation analysis, we observed that high Gad1 expressing lesion rats showed lower DNA methylation. Overall, Gad1 expression was highly variable across samples (also seen in Fig. 2b). One explanation is that Gad1 expression highly depends on neuronal activity, which has been described as a major regulator with a high temporal resolution [32–34]. Nevertheless, the epigenetic dysregulation in lesion animals shows the relevance of this mechanism.

The NAc is a crucial brain region mediating motivation and reward processing as well as social behavior [35–37]. NAc activity is mainly modulated by dopaminergic afferents from the ventral tegmental area and glutamatergic input from the PFC and thalamus. However, GABAergic inputs from these regions have been described as well [38–40]. Furthermore, GABA is the primary neurotransmitter of the inter- and projection neurons of the NAc [41]. As the integrative center of fastigial input via the ventral tegmental area, PFC, and thalamus, the internal processing of the NAc may be altered by lesions of the fastigial nucleus, leading to social and learning deficits as already reported in previous studies [11, 12]. It remains open why altered Gad1 methylation and expression were restricted to the NAc. Further experiments targeting the ventral tegmental area and more specific GABAergic markers may give further insight into this question.

Lesions of the fastigial nucleus also affected the oxytocinergic system. Besides its principal function as a hormone during childbirth, breastfeeding, and sexual reproduction, oxytocin also plays a vital role as a neurotransmitter in social behaviors and emotions [24, 25, 42, 43]. Among the most prominent Oxtr-expressing regions in the brain are the PFC and the NAc [43]. Its expression is regulated by a complex combination of direct oxytocin feedback as well as hormonal, inflammatory, and epigenetic factors [43]. As social dysfunctions are core symptoms of autism and schizophrenia, oxytocin dysregulation has been associated with these diseases [25, 44].

Here, we found one CpG position in the Oxtr gene promoter that was hypomethylated in lesion rats. In contrast, protein expression was highly variable across animals and showed no differences between experimental groups. The lack of correlation between these results may be explained by the influence of multiple regulating factors accompanied by the relatively small changes observed here.

Nevertheless, the altered epigenetic regulation of Oxtr in the PFC of rats with lesions of the fastigial nucleus strengthens the evidence for a fastigial contribution to emotional and social functions [11, 12]. Furthermore, as the cerebellar midline structures, including the fastigial nucleus, are discussed as a common source of autism and the cerebellar cognitive affecting syndrome, these findings are in line with reports on epigenetic modifications of Oxtr in autistic patients [45, 46].

Interestingly, overall changes were restricted to the NAc and the PFC, highlighting the importance of fastigial connections to circuits involving the PFC and the NAc [7–9]. Even though the dopaminergic system is affected by cerebellar lesions [47, 48], in the current study, we neither saw changes of promoter methylation in dopaminergic targets nor in glutamatergic targets (Drd2, Slc6a3, and Grin1). However, despite the long period between surgery and final analysis, we saw significant differences between experimental groups concerning epigenetic regulation of GABAergic and the oxytocinergic system after lesions of the cerebellar fastigial nucleus that may, at least in part, account for the non-motor deficits seen in this model.

## Conclusion

We showed that fastigial nucleus lesions in juvenile rats impact epigenetic regulation of the GABAergic and the oxytocin system as indicated by lower DNA methylation. Together with altered neuronal activity in the PFC that has been reported in this animal model, the long-term social, cognitive, and affective deficits have similarities to the cerebellar cognitive affecting syndrome [11, 12]. Our results, therefore, may promote insight into the clinical manifestation of cerebellar injuries on sociability, affect, and cognition, as seen in the cerebellar cognitive affective syndrome and other neuropsychiatric disorders like autism and schizophrenia.

## Supplementary Information

Below is the link to the electronic supplementary material.

Supplementary figure 1 Mean DNA methylation (%) for Drd2 (A), Slc6a3 (B), and Grin1 (C). Data is shown for lesion, sham-lesion, and control animals (box-plot: line – median, box limits – 1st and 3rd quartile, whiskers – min/max in 1.5 interquartile range) (PDF 150 KB)

Supplementary Table 1 List of primer sequences and fragments sizes of each target gene (XLSX 10 KB)

Supplementary Table 2 Detailed results of the ANOVA for methylation and protein expression (XLSX 10 KB)

Supplementary Table 3 List of transcription factor motives overlapping with respective CpG positions in the promoter region of Gad1 (XLSX 10 KB)
