# Supplementary figures and images for: Epigenetic Regulation of Neural Transmission after Cerebellar Fastigial Nucleus Lesions in Juvenile Rats
Source: Cerebellum. 2021 Apr 8;20(6):922–30. doi: 10.1007/s12311-021-01264-5 (PMC8674159; doi:10.1007/s12311-021-01264-5)

# A) DRD2

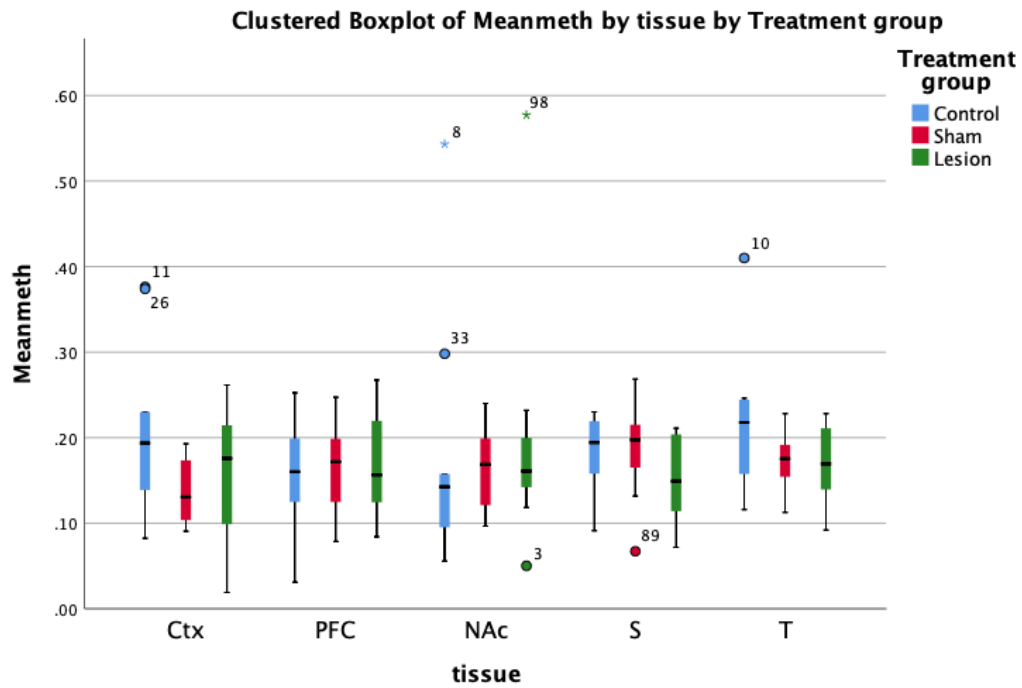

# B) DAT

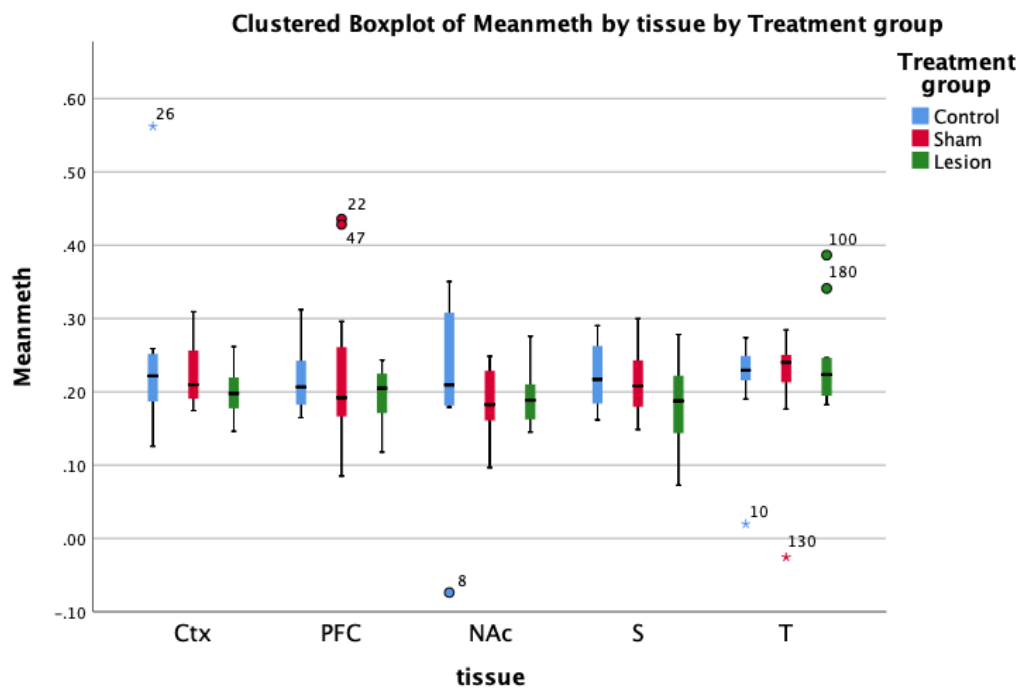

C) Grin1

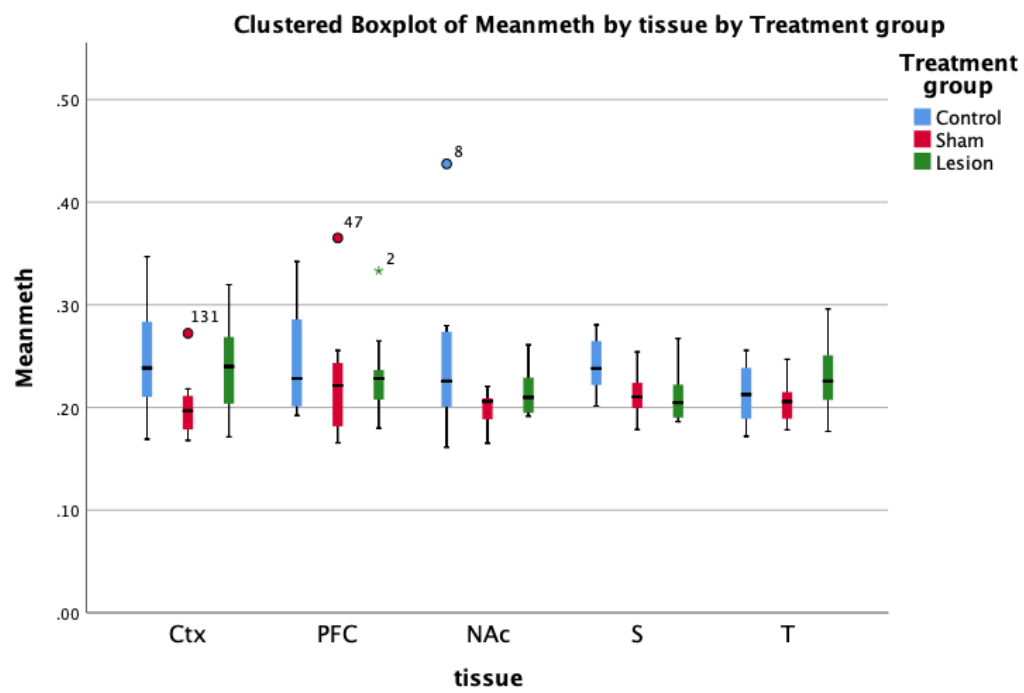

Supplement: Supplementary file 1 — Supplementary figure 1 Mean DNA methylation (%) for Drd2 (A), Slc6a3 (B), and Grin1 (C). Data is shown for lesion, sham-lesion, and control animals (box-plot: line – median, box limits – 1st and 3rd quartile, whiskers – min/max in 1.5 interquartile range) (PDF 150 KB) [file 12311_2021_1264_MOESM1_ESM.pdf]
